# A Rare Case of Coexisting Psychogenic Polydipsia and Nephrogenic Diabetes Insipidus With Lithium Therapy

**DOI:** 10.7759/cureus.23438

**Published:** 2022-03-24

**Authors:** Drashti Antala, Alisha Sharma, Arjab Adhikari, Pankaj Luitel, Sheldon Hirsch

**Affiliations:** 1 Internal Medicine, AMITA Health Saint Francis Hospital, Evanston, USA; 2 Nephrology, AMITA Health Saint Francis Hospital, Evanston, USA

**Keywords:** lithium-induced partial diabetes insipidus, polydipsia-polyuria syndrome, psychogenic polydipsia and lithium therapy, hypernatremia, hyponatremia, psychogenic polydipsia, nephrogenic diabetes insipidus

## Abstract

Lithium is a commonly used medication for mood stabilization and a well-known cause of nephrogenic diabetes insipidus (DI). Coexistent psychogenic polydipsia with nephrogenic DI is uncommon, and its management is challenging due to the wide variation in serum sodium based on fluctuations in water intake. Here, we describe the case of a 56-year-old male with psychogenic polydipsia and nephrogenic DI which manifested in wide swings of serum sodium over a short interval. He initially presented with hyponatremia with low urine osmolality consistent with psychogenic polydipsia. His serum sodium began to improve after free water restriction. However, later in the course, he developed an increase in serum sodium levels and polyuria with persistent low urine osmolality consistent with DI.

## Introduction

Psychogenic polydipsia is a well-known phenomenon characterized by excessive fluid intake and polyuria. It is found in 11-20% of patients with schizophrenia spectrum disorder [1]. Lithium is a medication used for mood stabilization in many psychiatric disorders. Nephrogenic diabetes is a known adverse effect associated with lithium therapy. Underlying nephrogenic diabetes insipidus (DI) predisposes patients to urinary water loss and hypernatremia unless patients replace the water loss with an increase in fluid intake. However, in the event of coexisting psychogenic polydipsia, patients can consume enough water to overcome the kidney’s ability to excrete it which can result in hyponatremia. Therefore, a patient with both psychogenic polydipsia and nephrogenic DI can develop either hypernatremia or hyponatremia or swing rapidly from one to the other depending on access to and intake of water.

## Case presentation

**Figure 1:**
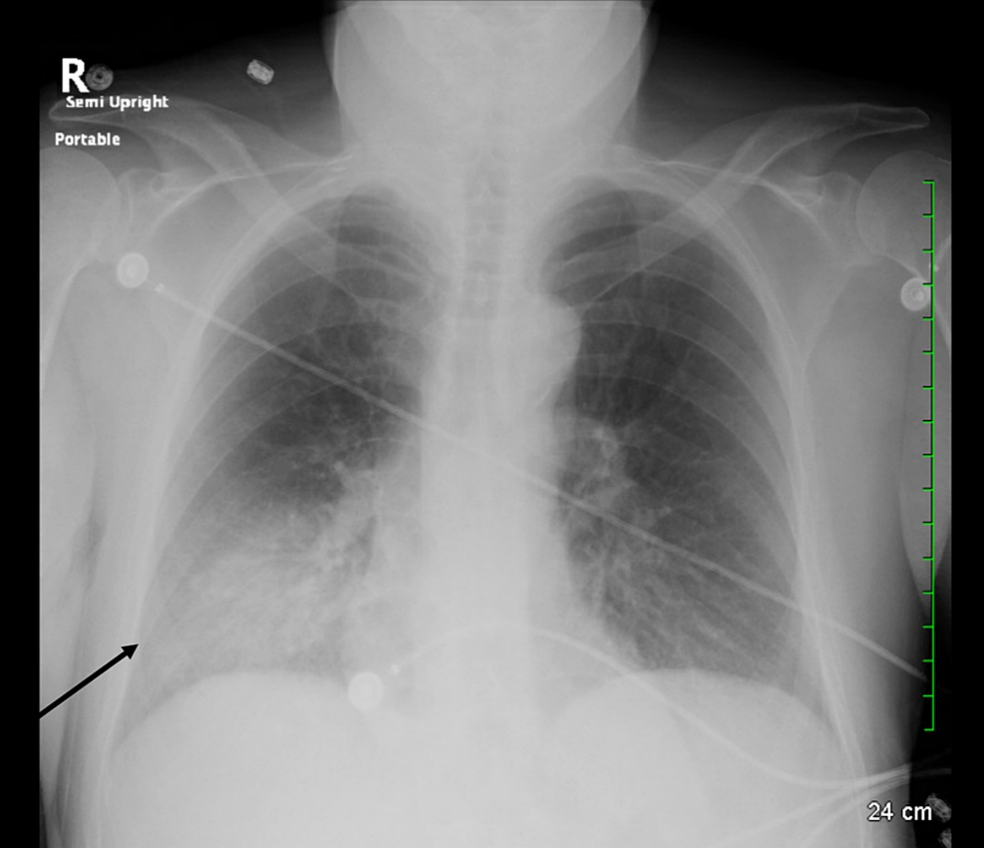
Chest X-ray showing right lower lobe infiltrate consistent with pneumonia.

A 56-year-old male with a history of schizoaffective disorder (diagnosed 20 years ago) presented to the Emergency Department with generalized weakness, fatigue, and fever for a day. His home medications included lithium, amitriptyline, and valproic acid. His vital signs were stable except for a temperature of 103.2°F. A physical examination was remarkable for crackles on the right lower region of the chest. A chest X-ray revealed an infiltrate in the right lower lobe for which he was started on broad-spectrum antibiotics amoxicillin/clavulanate and doxycycline (Figure 1). Urine antigens for *Legionella* and *Streptococcus pneumoniae* were negative, and mycoplasma immunoglobulin M was not elevated. Labs were remarkable for serum sodium of 127 mmol/L and serum creatinine of 1.48 mg/dL. Serum osmolality was 271 mOsm/kg (reference range: 275-295 mOsm/kg), and urine osmolality was 83 mOsm/kg (reference range: 50-1,200 mOsm/kg) which in the presence of hyponatremia suggested a diagnosis of psychogenic polydipsia. The patient was placed on fluid restriction of 1 L. Repeat labs in eight hours showed sodium of 133 mmol/L and creatinine of 1.08 mg/dL. The patient had a urine output of 2,700 cc over 24 hours, and the next day sodium corrected to 142 mmol/L with fluid restriction. Urine output subsequently increased to 4,400 cc in 24 hours and sodium increased to 145 mmol/L. Once his sodium level increased, his fluid restriction was stopped. He continued to have polyuria with urine output on average 5.5 L/24 hours for the next few days. At this time, despite the rise in serum sodium, his urine osmolality remained low at 103 mOsm/kg which was consistent with DI due to long-standing lithium use. He then maintained serum sodium in the high normal range because of spontaneous water intake. During hospitalization, he underwent informal water deprivation by being started on fluid restriction after which he developed a rise in sodium level and polyuria with low urine osmolality, essentially consistent with DI.

## Discussion

Lithium is one of the oldest mood stabilizers and was approved in 1970 by the Food and Drug Administration [2]. It remained the first line of management for many years. However, its use has been declining due to its toxicity and narrow therapeutic window [2]. Lithium is not protein-bound and is freely distributed throughout the body. It is not metabolized, and almost 95% of it is excreted unchanged through the kidney [3]. The plasma elimination half-life of a single dose ranges from 18 to 20 hours in young adults to approximately 36 hours in elderly patients. The half-life is also affected by the duration of therapy, increasing with the duration the patient has been on the treatment [4].

Because lithium has the same charge as sodium, its mechanism of filtration and reabsorption is very similar to sodium. It is freely filtered in the kidney, and 70% of it is reabsorbed in the proximal tubule. However, compared to sodium, lithium is less absorbed and delivered more to the distal part of the nephrons [5]. Overall, 3-10% of lithium is absorbed in the loop of Henle while the remaining is reabsorbed by transcellular uptake via epithelial sodium channel in the distal tubule and collecting duct [6,5]. The serum lithium concentration may not correlate with toxicity. Around 40% of patients who receive lithium therapy develop nephrogenic DI [7], which can sometimes persist for years after discontinuing lithium [8], even becoming irreversible after chronic use [9].

Nephrogenic DI is characterized by an inability of the kidneys to concentrate urine in the presence of antidiuretic hormone causing polyuria [10]. The aquaporin-2 water channel (AQP2) is expressed in the principal cells and plays an essential role in the reabsorption of water in the collecting ducts via the type 2 vasopressin receptor (V2R)-mediated mechanism. The dysfunction of the AVP-V2R-AQP2 system can result in DI [11]. Lithium prevents the insertion of cytoplasmic urinary aquaporin (AQP2) to the apical membrane because of which more hypo-osmotic fluid is delivered to the medullary collecting duct resulting in large-volume dilute urine excretion [12]. An open clinical trial with amiloride showed that impaired concentrating ability in lithium-induced nephrogenic DI was associated with decreased urinary aquaporin excretion, and this association correlated with the duration of lithium exposure [12].

Our patient had been on lithium for a long time and may have developed chronic nephrogenic DI. The diagnosis was obscured on admission due to hyponatremia. Nephrogenic DI manifested only after water restriction was enforced. He underwent an informal water deprivation test by being started on fluid restriction, after which the serum sodium increased to 145 mEq/L while the urine osmolality remained low (103 mOsm/kg), which is essentially diagnostic of DI. Polyuria tends to decrease in patients with primary polydipsia after eight hours of water deprivation and urine osmolality increases, whereas in DI, polyuria does not improve after water deprivation and serum osmolality remains low, as seen in this patient. The desmopressin test was not done due to concerns regarding cost and clinical correlation. As he was on lithium for a long duration, it was likely nephrogenic DI. Another case has been described of a patient with both psychogenic polydipsia and lithium-induced nephrogenic DI [13]. Further studies are needed to study whether lithium has any effect on the thirst mechanism and a role in developing primary polydipsia.

Polyuria-polydipsia syndrome describes a clinical syndrome of excessive intake and urinary output, with the usual differential diagnosis including psychogenic polydipsia and central or nephrogenic DI [14]. In rare cases, when polydipsia and nephrogenic DI coexist, multiple tests are needed at different times in the clinical course to provide a complete analysis. The water deprivation test is the best diagnostic test to differentiate among the different etiologies [1]. Studies have also found that copeptin, the c-terminal part of the AVP precursor peptide, can serve as a sensitive surrogate marker for AVP. Copeptin has been shown to improve the diagnostic accuracy of water deprivation test and help discriminate between primary polydipsia and DI [1]. Further studies are needed to accurately determine the objective way of differentiating between the two entities.

## Conclusions

Having psychogenic polydipsia and diabetes insipidus at the same time can be challenging to manage due to wide fluctuations in sodium with changes in water intake, and can even prove catastrophic if sodium drops or rises to extreme levels. Because psychogenic polydipsia is widely prevalent in psychiatric patients and many patients are on lithium therapy, this condition may be more common, though very few cases have been reported. Further studies are needed to study this and explore the pathophysiology behind the possible role of lithium in the development of psychogenic polydipsia.
